# Long Noncoding RNA AC007639.1 Promotes the Pathogenesis and Progression of Hepatocellular Carcinoma Through Inhibiting Apoptosis and Stimulating Chemotherapeutic Resistance

**DOI:** 10.3389/fonc.2021.715541

**Published:** 2021-09-02

**Authors:** Yun Bai, Meijuan Ding, Dan Lu, Yiwen Li, Shuai Yao, Lei Wang, Hui Li, Guanghua Cui, Xue Li, Xiaoke Sun, Yu Yang

**Affiliations:** ^1^Department of Oncology, Second Affiliated Hospital of Harbin Medical University, Harbin, China; ^2^Department of Oncology, Harbin Medical University Cancer Hospital, Harbin, China; ^3^Department of Technology, Harbin Nachuan Bio-Science Technology Co., Ltd., Harbin, China; ^4^Department of Internal Medicine, Second Hospital of Heilongjiang Province, Harbin, China

**Keywords:** long noncoding RNA, hepatocellular carcinoma, bioinformatics, p53, chemotherapy

## Abstract

**Background:**

Hepatocellular carcinoma (HCC) is known for its poor prognosis. Long noncoding RNAs (lncRNAs) are critical in the pathogenesis of various types of cancers. We tried to explore the role of lncRNA in the development of HCC.

**Methods:**

We identified the role of lncRNA AC007639.1 in the pathogenesis of HCC through bioinformatics and biological experiments in HepG2, Hep3B, and SMMC-7721 cells as well as the nude mice xenograft model.

**Results:**

We found that lncRNA AC007639.1 was overexpressed in hepatocellular carcinoma. Knocking down of lncRNA AC007639.1 by specific siRNAs or shRNAs promoted cancer cell death. The growth of mouse xenograft tumor created using lncRNA AC007639.1 deficient HepG2 cells was significantly slowed down. Furthermore, the knockdown of lncRNA AC007639.1 in HCC cells led to the increased expression of p53 and decreased expression of angiopoietin-like 4.

**Conclusion:**

LncRNA AC007639.1 was involved in the pathogenesis and progression of hepatocellular carcinoma by inhibition of apoptosis and increasing HCC resistance to chemotherapy.

## Introduction

The American Cancer Society estimates that there will be about 42,230 new cases of liver cancer, with 30,230 deaths due to liver cancer in the United States in 2021, representing a more than tripled incidence and more than doubled mortality since 1980. Worldwide, people diagnosed with liver cancer exceed 800,000 and more than 700,000 deaths are due to liver cancer each year, making liver cancer a leading cause of cancer-related deaths (1). One of the main causes of liver cancer, hepatitis B virus infection, is even more deleterious in leading to fulminant hepatitis (2–5), and the virus can be passed to the newborns without proper control of HBV level and HBV vaccination (6). Therefore, the mechanisms of tumorigenesis and factors affecting the prognosis of hepatocellular carcinoma (HCC) deserve more attention.

Long noncoding RNAs (lncRNAs), by definition, are not translated into proteins (7). Increasing evidence has shown that lncRNAs are involved in the pathogenesis and progression of different types of cancers (8, 9). Many of the lncRNAs are promising markers for the diagnosis and prognosis of liver cancer (10).

In this study, the role of lncRNA AC007639.1 in the pathogenesis and prognosis of HCC was explored by bioinformatics analysis and biological experiments using three different liver cancer cell lines. We found that lncRNA AC007639.1 knockdown led to increased cancer cell death. Importantly, lncRNA AC007639.1 could inhibit the p53 signaling pathway and increase the expression of Angiopoietin-like 4 (ANGPTL4) leading to inhibition of apoptosis and increasing of HCC resistance to chemotherapy.

## Materials and Methods

### Bioinformatic Analysis

Transcriptome sequencing data including mRNA and lncRNA were generated by R package in Liver Hepatocellular Carcinoma Project of The Cancer Genome Atlas (TCGA-LIHC) dataset (n=420) using TCGA biolinks (https://bioconductor.org/packages/release/data/experiment/vignettes/TCGAbiolinksGUI.data/inst/doc/vignettes.html), and SRP069212 (n=355) using Gene Expression Profiling Interactive Analysis (GEPIA; http://gepia.cancer-pku.cn/). Differential mRNA abundance was analyzed using DESeq2 (11). Genes with reads > 5 were included in the final quantitative and statistical analysis. Heatmaps and volcano plots were prepared using the R package. Normalized gene expression levels were analyzed by Gene Set Variation Analysis (GSVA). Survival analysis was done by R package survival. Cox proportional hazard (PH) model was constructed by R package (survminer). The best-scanned cutoff points had the most significant split (log-rank test).

### Cell Culture

Human HCC cell lines HepG2 (RRID : CVCL_ 0027), SMMC-7721 (RRID : CVCL_ 0534), and Hep3B (RRID : CVCL_ 0326), were obtained from the Shanghai Zhong Qiao Xin Zhou Biothechnology Co., Ltd., (Shanghai, China). The cells were confirmed to have no mycoplasma contamination using Mycoplasma Detection Kit (R&D Systems China Co., Ltd., Shanghai, China). Dulbecco’s modified Eagle’s medium (DMEM, Gibco, Thermo Fisher Scientific, Inc.) was used for cell culture, with 10% fetal bovine serum (FBS, Gibco), 2 mM L-glutamine (Gibco), 10 mM HEPES (Gibco), 1 mM pyruvate sodium (Gibco), and 100 U/ml penicillin with 100 µg/ml streptomycin (Gibco). Cells were grown at 37°C with 5% CO_2_.

### Construction of Stable Knockdown of Lnc RNA AC007639.1 in HepG2 and Hep3B Cells

Cells with stable lnc RNA AC007639.1**-**knockdown, ANGPTL4-knockdown, or control HepG2-LNC-NC cells were created using shRNA 5’- GGUGAGUGCAUGUAGUCAUTT -3’, 5’- AGAACAGCAGGAUCCAGCAACUCUU -3’, or scramble control sequence 5’- UUCUCCGAACGUGUCACGUTT -3’. shRNA oligos were cloned into a LV3(H1/GFP&Puro) vector (map shown in **Figure S1**), respectively. The corresponding plasmids were packaged using lentivirus (Shanghai GenePharma, China). Puromycin (5 μg/ml final concentration, Sigma, St. Louis, MO) was used to select HCC cells carrying the transfected shRNA.

### siRNAs Transfection Into HCC Cells

Specific short interfering RNAs (siRNAs) or control siRNA to knock down lnc RNA AC007639.1 were purchased from the Shanghai GenePharma (Shanghai, China). 30nM of siRNAs (**Table S1**) were used for transfection in the HCC cells using X-tremeGENE reagent (Roche Applied Science, Shanghai, China).

### RNA Extraction and Quantitative RT-PCR

For RNA extraction, cells or tissues were homogenized with TRIzol reagent (Thermo Fisher Scientific, Inc.). RNAs were purified and quantified using the NanoDrop 2000 Spectrophotometer (Thermo Fisher Scientific, Inc.). RNAs were reversely transcribed by the RT reagent Kit (Nachuan Bio-Tech Co., Binzhou, China). qRT-PCR experiments were done in 10 µL total volume, which contained 1x SYBR Green Master mix (Nachuan Bio-Tech Co., China), cDNA (10 ng), and primers (75 nM of forward and 75 nM reverse primers, **Table S1**) in an Exicycle 96 Real-Time Quantitative Thermal Block (Bioneer, China), with initial incubation at 95°C (10 min), 40 cycles at 95°C (15 s) and 60°C (1 min). qRT-PCR experiments were triplicated, the averages of which were normalized (by β-actin), and the relative expression of AC007639.1 was calculated using the 2^−ΔΔCt^ method.

### Cell Count Kit-8 Assay

Cell proliferation was determined using the CCK-8 assay kit (DOJINDO, Japan). Briefly, cells (1 x 10^4^/well) were grown in 96-well plates. At the same time of each day (10 am), CCK-8 reagent (10 μl) was diluted with 100 μl medium and added to each well. After incubation for 2 hours, the light absorbance at 450 nm was recorded with a microplate spectrophotometer (K8001, Shanghai Yoke Instrument Co., Ltd., China) (12).

### EdU Assay

The EdU assay was performed using a KFluor488 Click It 5-ethynyl-2’-deoxyuridine Imaging Test Kit (KGA331-500; keyGen, Nanjing, China). At 24 hours after transfection, 10^5^ cells were seeded in each well of a 24-well plate. After incubation for 24 hours, EdU was added (final concentration of 50 µM). After 2 hours incubation with EdU, cells were fixed with 150 µl of 4% formaldehyde in PBS for 20 min. A 495-nm laser was used to excite kFluor488-azide and images were captured under a fluorescence microscope (IX81, Olympus Corporation, Beijing, China). Nuclei were counterstained with DAPI.

### Western Blotting

Forty-eight hours post transfection, cells were lysed in RIPA lysis buffer (Merck Group, Germany) for 30 min on ice. Protein concentrations were determined using a BCA assay kit (Solarbio, China). Lysates with equal amounts were loaded and separated by 12% SDS-polyacrylamide gel electrophoresis. Proteins were transferred onto polyvinylidene difluoride membranes, followed by probing with target antibodies. Primary antibodies included: Beta-actin (Affinity T0022, 1:1000), and p53 (Affinity AF0879, 1:1000), and the secondary antibody (Affinity S0001, 1:5000) were obtained from Xiangtai Biological Technology Co., Ltd. (China); and ANGPTL4 (Boster A01147, 1:1000) was purchased from Boster Biological Technology (Pleasanton, CA).

### Establishment of Mouse Xenograft Tumor Model

The animal protocol was approved by the Ethical Committee of the Second Affiliated Hospital of Harbin Medical University (a tertiary hospital in northeast China). Male BALB/c nude mice were purchased from Beijing Vital River Laboratory Animal Technology Co., Ltd., and housed in a temperature-controlled, specific-pathogen-free animal facility, with a 12h light/12h dark cycle and free access to food and water. Animals were properly treated in accordance with the national and institutional ethical requirements of experimental animals. 1.5 × 10^6^ of HepG2-LNC-KD or HepG2-LNC-NC cells were resuspended in 0.1 ml sterile PBS, and subcutaneously injected in the left flank of mice at the age of 8 weeks. The size of tumor was monitored every morning (length x width x depth in mm^3^). Mice were sacrificed 2 weeks after cell injection (n = 7 per group).

### Immunohistochemistry

Sections (10 μm) of paraffin-embedded xenograft tissue samples were used for immunohistochemistry staining. Slides were incubated with PCNA antibody (1:100 dilution in PBS; AF0239, Affinity Biosciences LTD.) or p53 (1:100 in PBS; Affinity AF0879) at overnight 4°C. After gentle rinsing off primary antibody solutions, slides were incubated with the secondary antibody (1:200 dilution in PBS; S0001, Affinity Biosciences LTD.) at 37°C for 1 hour. Finally, nuclei were counterstained with hematoxylin.

### Statistics

Except for bioinformatics, statistical analyses were done with SPSS version 24.0 (Armonk, NY: IBM Corp.). Continuous data were shown as mean ± SD. Differences between the two groups were analyzed by independent Student’s t-test. p < 0.05 was considered significant in 2-tailed statistical tests.

## Results

### LncRNA AC007639.1 Expression in HCC

**Figure 1:**
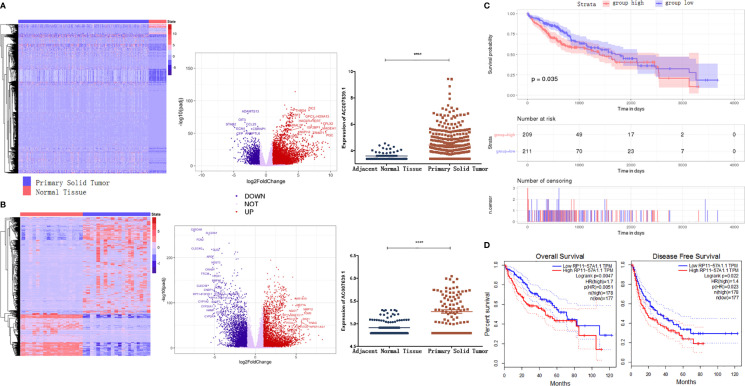
LncRNA AC007639.1 is highly expressed in hepatocellular carcinoma tissues and implies a poor prognosis. **(A)** Heatmap of transcriptome (left) and volcano plot (middle) of different genes, and lncRNA AC007639.1 expressions in TCGA-LIHC cases (right, n=420, https://gdc.cancer.gov/resources-tcga-users/tcga-code-tables/data-levels); **(B)** Heatmap of transcriptome (left) and volcano plot (middle) of different genes, and lncRNA AC007639.1 expressions in SRP069212 cases (right, n=355); **(C, D)** Kaplan-Meier survival curves of TCGA-LIHC dataset analyzed by TCGAbiolinks or SRP069212 dataset analyzed by GEPIA, respectively. ****p < 0.001 (Mann-Whitney test).

According to the bioinformatic analysis of TCGA-LIHC dataset using the DESeq2 software package, lncRNA AC007639.1 expression in HCC tissues was found to be significantly higher than that of adjacent non-tumor liver tissue (**Figures 1A, B**). The median level of lncRNA AC007639.1 was used as a cut-off to differentiate the high- and low- AC007639.1 expression groups. Using the GEPIA online tool (13) and R language survival analysis, the patients in the high AC007639.1 expression group were found to have a shorter survival period (**Figures 1C, D**). These results indicated that AC007639.1 was overexpressed in HCC tissues and the high level of AC007639.1 was indicative of poor prognoses.

### Prediction of the Functions of LncRNA AC007639.1 in HCC

**Figure 2:**
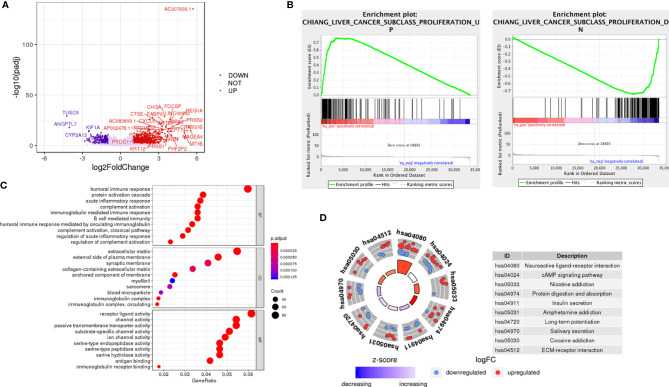
The role of LncRNA AC007639.1 in liver cancer. **(A)** Differential gene expressions between the high- and low- expression of lncRNA AC007639.1 groups (TCGA-LIHC). **(B)** LncRNA AC007639.1 functions analyzed by GSVA. The up-regulated genes in proliferative liver cancer were highly enriched in the AC007639.1 high expression group (p<0.001), while the down-regulated genes in proliferative liver cancer is highly enriched in the AC007639.1 low expression group (p<0.001). **(C)** GO analysis and **(D)** KEGG and cluster network analysis using Clusterprofiler Package of R language, screening criteria p < 0.05, correction p < 0.05.

**Figure 3:**
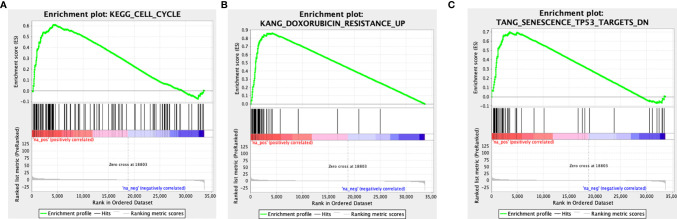
LncRNA AC007639.1 regulates the susceptibility of liver cancer to doxorubicin by inhibiting the p53 pathway, analyzed by GSVA. **(A)** The effect of lncRNA AC007639.1 on cell cycle (p < 0.001); **(B)** The effects of lncRNA AC007639.1 on doxorubicin resistance (p < 0.001); **(C)** The effects of lncRNA AC007639.1 on p53 target genes (p < 0.001).

Differentially expressed genes in the high- and low- AC007639.1 expression groups in the TCGA-LIHC dataset were analyzed by DEseq2 (**Figure 2A**). The enrichment of genes was analyzed using GSVA. The AC007639.1 high expression group was found to have highly enriched HCC up-regulating gene sets (**Figure 2B**). GO enrichment analysis (14) showed that AC007639.1 was involved in diverse cell functions such as immune functions (**Figures 2C, D**). LncRNA AC007639.1 was found to regulate the cell cycle (**Figure 3A**), increase HCC resistance to Doxorubicin (**Figure 3B**), and decrease the protein expression of p53 (**Figure 3C**).

### LncRNA AC007639.1 in the Proliferation of HCC Cells

**Figure 4:**
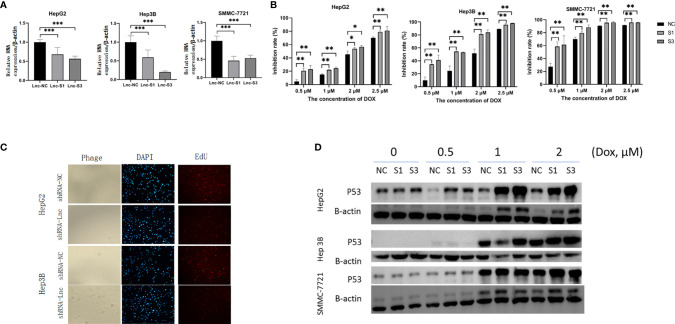
Knockdown of lncRNA AC007639.1 inhibits the growth of hepatocellular carcinoma cells and promotes apoptosis. **(A)** qRT-PCR on the relative AC007639.1 expression in HepG2, Hep3B, and SMMC-7721 cells transfected with two lncRNA AC007639.1 or control siRNAs (Lnc-S1, Lnc-S3, or Lnc-NC, respectively); **(B)** CCK-8 assay showing inhibition of HepG2, Hep3B, and SMMC-7721 cells at different concentrations of doxorubicin after siRNAs transfection; **(C)** EdU analysis showing cell proliferation after transfection of scrambled shRNA (NC) or shRNA for lncRNA (shRNA-Lnc) (field of view: 200x) in HepG2 or Hep3B cells treated with 2.5 µM doxorubicin for 48 hours; **(D)** Western blots of p53 in the three cells lines after exposure to different concentrations of doxorubicin. NC: scrambled control siRNA or shRNA. S1, siRNA1; S3, siRNA3. *p < 0.05, **p < 0.01, ***p < 0.001.

In order to investigate the function of AC007639.1 in HCC, three specific siRNAs were designed to knock down its expression in HCC cell lines, among which both siRNA1 and siRNA3 significantly downregulated AC007639.1 in HepG2, Hep3B, and SMMC-7721 cells (p<0.001 for both siRNAs, **Figure 4A**). The lncRNA AC007639.1 knockdown cells showed higher inhibition of proliferation after treated with different concentrations of Doxorubicin than that of the control groups (**Figure 4B**), which was also confirmed by EdU analysis (**Figure 4C**). Expression of p53 protein in HepG2, Hep3B, and SMMC-7721 cells were increased when treated with DOX after siRNA1 and siRNA3 transfection (**Figure 4D**). The above findings suggested that knocking down of AC007639.1 inhibited HCC cell growth, and promoted HCC apoptosis.

### LncRNA AC007639.1 in Nude Mice Xenografts

**Figure 5:**
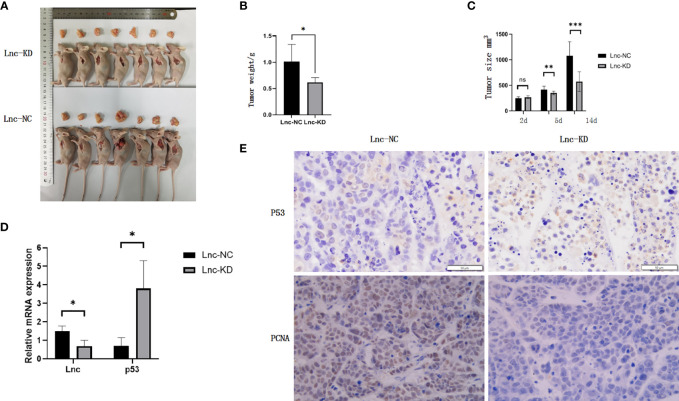
Knockdown of lncRNA AC007639.1 inhibits the growth of tumor xenografts in nude mice. **(A)** The size of HepG2 xenografts using HepG2-LNC-KD or HepG2-LNC-NC cells at 14 days; **(B)** Weight of xenografts at collection; **(C)** Xenografts growth monitored in mm^3^; **(D)** Relative AC007639.1 and p53 expressions (normalized to β-actin) in xenografts with HepG2-LNC-KD or HepG2-LNC-NC cells; **(E)** p53 and PCNA immunohistochemistry in xenograft tissues (field of view: 200x). Brown colored cells were positive for P53 or PCNA, respectively. *p < 0.05, **p < 0.01, ***p < 0.001; ns, not statistically significant.

To explore the role of AC007639.1 *in vivo*, we developed the HepG2-LNC-KD cells that carried a stable knockdown level of AC007639.1 (confirmed by qRT-PCR, data not shown), and used the cells to create a xenograft tumor mouse model in nude mice. The size of xenograft tumor was significantly smaller in the stable knockdown group at 2 weeks compared with the knockdown group (**Figures 5A, B**), with a slower time-course growth rate (**Figure 5C**). The expression of AC007639.1 in the xenograft were significantly lower in the knockdown group, with a significant higher P53 expression (**Figure 5D**). P53 and PCNA immunohistochemistry showed increased p53-positive cells, reduced cell atypia, and reduced PCNA-positive cells after AC007639.1 was knocked down (**Figure 5E**). These results showed that lncRNA AC007639.1 is important in xenograft tumor growth.

### The Mechanism of AC007639.1 in Regulating Cell Functions

**Figure 6:**
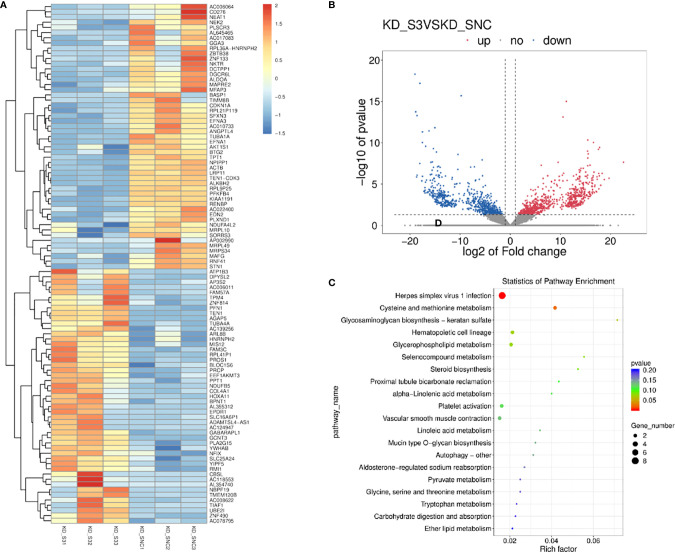
The involvement of lncRNA AC007639.1 in different pathways. **(A)** Heatmap showing different gene expressions after the knock down of lncRNA AC007639.1 in HepG2 cells using siRNA3; **(B)** Volcano plot of different gene expressions after the knock down of lncRNA AC007639.1 in HepG2 cell line; **(C)** Pathways enrichment after the knock down of lncRNA AC007639.1 in HepG2 cell line.

**Figure 7:**
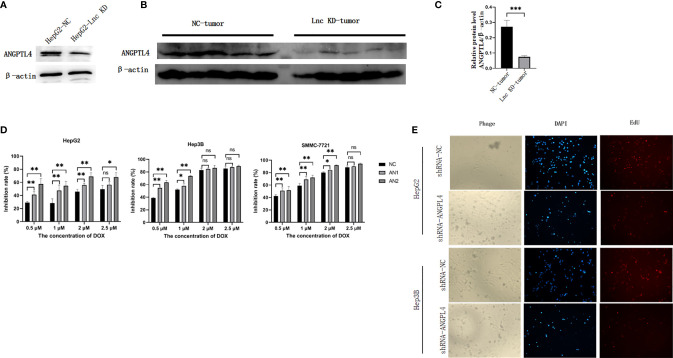
The role of ANGPTL4 in hepatocellular carcinoma cells. **(A)** The protein expression of ANGPTL4 in HepG2 cells after transfection of NC siRNA or siRNA3 (n=5). Protein **(B)** or RNA **(C)** expression of ANGPTL4 in HepG2 xenografts. **(D)** Knocking down of ANGPTL4 using ANGPTL4-siRNA 1 (AN1) or ANGPTL4-siRNA 2 (AN2) increased the inhibition of Doxorubicin in HepG2, Hep3B, and SMMC-7721 cells. **(E)** EdU analysis showing cell proliferation after transfection of scrambled shRNA (shRNA-NC) or shRNA-ANGPTL4 (field of view: 200x) in HepG2 or Hep3B cells treated with 2.5 µM doxorubicin for 48 hours. *p < 0.05, **p < 0.01, ***p < 0.001; ns, not statistically significant.

The above bioinformatics analysis indicated that AC007639.1 regulated HCC cell proliferation. We further analyzed the role of lncRNA AC007639.1 by sequencing gene expressions in HepG2 (**Figures 6A–C**) cells after the transfection of siRNA3. Among the genes with significant changes, ANGPTL4 was selected for further analysis. ANGPTL4 protein was significantly lower in HepG2 cells after AC007639.1 was knocked down (**Figure 7A**), and ANGPTL4 protein level also decreased in xenografts injected with HepG2-LNC-KD (**Figures 7B, C**). Knocking down of ANGPTL4 increased the inhibitory effect of Doxorubicin in HepG2, Hep3B, and SMMC-7721 cells (**Figures 7D, E**).

## Discussion

LncRNAs and endonuclease (15) are involved in the pathogenesis of liver cancer. Most lncRNAs promote the proliferation of HCC through microRNAs (16).

The application of bioinformatic analyses facilitated the identification of candidate lncRNAs and related signaling pathways for indispensable mechanistic studies. According to the findings from the TCGA-LIHC dataset, lncRNA AC007639.1 was knocked down in three HCC cell lines using specific siRNAs or shRNA, with the finding of significantly more cell death and inhibition of cell proliferation, which showed lncRNA AC007639.1 is involved in HCC pathogenesis.

We tested the role of lncRNA AC007639.1 in three cell lines of hepatic origin, HepG2, Hep3B, and SMMC-7721. HepG2 is characterized by hepatitis B virus negative and non-tumorigenic, while Hep3B is characterized by hepatitis B virus positive and tumorigenic (17), and SMMC-7721 has been suspected of its liver origin due to contamination concerns (18). Therefore, to avoid controversies, SMMC-7721 cells were just used to test the efficacy of siRNA knock down (19), but not selected for further mechanistic experiments. Doxorubicin is among the most used chemotherapeutics against human cancers (20), the resistance of which is related with lncRNA AC007639.1 as shown in this study.

ANGPTL4 protein belongs to the angiopoietin (ANG)-related family. It is highly expressed in numerous organs including liver, and is be stimulated by inflammatory or hypoxic conditions (21, 22). However, the roles of ANGPTL4 in human cancers are controversial in different experimental models and proposed pathways. Overexpression of ANGPTL4 promotes tumorigenesis and metastasis (23), whereas it presents anti-metastatic activity through inhibition of vascular permeability and invasiveness (24). In the clinical settings, a high serum ANGPTL4 protein level in HCC patients is predictive of liver cirrhosis and intrahepatic metastasis (25). But levels of ANGPTL4 protein in tumor tissues are significantly lower than that in non-tumor tissues of the same HCC patients (26). Knockdown of ANGPTL4 inhibits the development of human gastric cancer (27). Our findings showed that the expression of ANGPTL4 is controlled by lncRNA AC007639.1, and ANGPTL4 contributed to the resistance of HCC cells to doxorubicin.

Taken together, we showed by bioinformatics and mechanistic studies that lncRNA AC007639.1 was involved in the pathogenesis of HCC by decreasing apoptosis and increasing resistance to chemotherapy. LncRNA AC007639.1 could be a valuable prognostic predictor as well as treatment target in HCC patients.

## Data Availability Statement

The datasets presented in this study can be found in online repositories. The names of the repository/repositories and accession number(s) can be found below: (https://www.ncbi.nlm.nih.gov/genbank/), PRJNA716423.

## Ethics Statement

The animal study was reviewed and approved by Ethical Committee of Second Affiliated Hospital of Harbin Medical University.

## Author Contributions

YY and YB conceived the study, analyzed and interpreted patient data, and were major contributors in writing the manuscript. YB, MD, DL, YL, SY, LW, HL, GC, XL, and XS obtained experiment data. All authors contributed to the article and approved the submitted version.

## Conflict of Interest

Author SY was employed by the company “Harbin Nachuan Bio-Science Technology Co., LTD.”.

The remaining authors declare that the research was conducted in the absence of any commercial or financial relationships that could be construed as a potential conflict of interest.

## Publisher’s Note

All claims expressed in this article are solely those of the authors and do not necessarily represent those of their affiliated organizations, or those of the publisher, the editors and the reviewers. Any product that may be evaluated in this article, or claim that may be made by its manufacturer, is not guaranteed or endorsed by the publisher.

## Supplementary Material

Supplementary Figure 1 The map of shRNA vector.

Supplementary Table 1 Primers for qRT-PCR and siRNAs.
