# Supplementary figures and images for: Long Noncoding RNA AC007639.1 Promotes the Pathogenesis and Progression of Hepatocellular Carcinoma Through Inhibiting Apoptosis and Stimulating Chemotherapeutic Resistance
Source: Front Oncol. 2021 Sep 2;11:715541. doi: 10.3389/fonc.2021.715541 (PMC8443795; doi:10.3389/fonc.2021.715541)

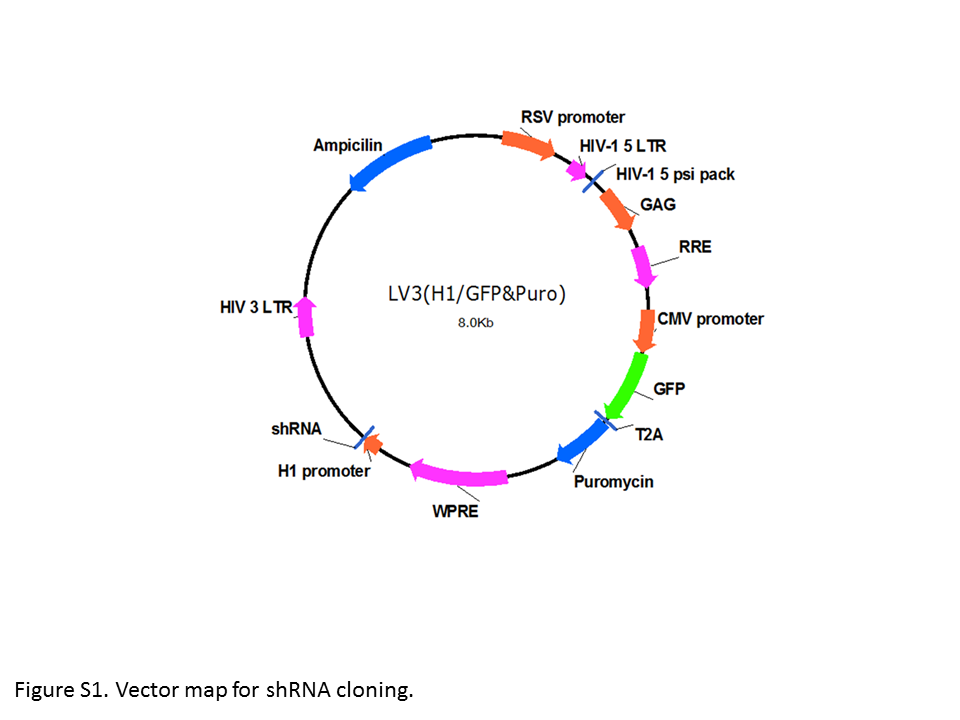

Supplement: Supplementary Figure 1 — The map of shRNA vector. [file Image_1.tif]
