# Supplementary material for: Long Noncoding RNA AC007639.1 Promotes the Pathogenesis and Progression of Hepatocellular Carcinoma Through Inhibiting Apoptosis and Stimulating Chemotherapeutic Resistance
Source: Front Oncol. 2021 Sep 2;11:715541. doi: 10.3389/fonc.2021.715541 (PMC8443795; doi:10.3389/fonc.2021.715541)
Supplement: Supplementary Table 1 — Primers for qRT-PCR and siRNAs. [file Table_1.docx]

**Table S1. Primer and oligonucleotide sequences for qRT-PCR and siRNAs.**

|  | qRT-PCR | 5’ to 3’ |
| --- | --- | --- |
| AC007639.1 | Forward | GCGGGGGATGAAAGAGATCC |
|  | Reverse | TACCTGCCCACCATCTCACT |
| ANGPTL4 | Forward | AGACACAACTCAAGGCTCAG |
|  | Reverse | CTCATGGTCTAGGTGCTTGTG |
| β-actin | Forward | CCATCGTCCACCGCAAAT |
|  | Reverse | GCTGTCACCTTCACCGTTCC |
|  | Lnc or gene |  |
| siRNA-1 | AC007639.1 | sense: GCUCAAUAAACAUCAGCUATT  antisense: UAGCUGAUGUUUAUUGAGCTT |
| siRNA-3 | AC007639.1 | sense: GGUGAGUGCAUGUAGUCAUTT  antisense: AUGACUACAUGCACUCACCTT |
| siRNA-AN1 | ANGPTL4 | sense: CAGACACAACUCAAGGCUCAGAACA  antisense: UGUUCUGAGCCUUGAGUUGUGUCUG |
| siRNA-AN2 | ANGPTL4 | sense: AGAACAGCAGGAUCCAGCAACUCUU  antisense: AAGAGUUGCUGGAUCCUGCUGUUCU |
| siRNA-NC | Non mammal | sense : UUCUCCGAACGUGUCACGUTT  antisense: ACGUGACACGUUCGGAGAATT |
